# Knowledge, attitude, and practice of e-cigarette use among undergraduate students: A comparative study between China and Indonesia

**DOI:** 10.18332/tid/190636

**Published:** 2024-07-16

**Authors:** Ronald Hartono, Chaofang Yan, Ying Chen, Boting Ma, Yaqi Deng, Yijia Sun, Pan Li, Yuye Dao, Rui Deng

**Affiliations:** 1 Kunming Medical University, Kunming, China

**Keywords:** knowledge, attitude, practice, e-cigarettes, China, Indonesia

## Abstract

**INTRODUCTION:**

The health risks associated with e-cigarettes are currently the focus of tobacco control efforts and public health initiatives. Given that China and Indonesia have the highest rates of adult smoking worldwide, it is imperative to gain a comprehensive understanding of e-cigarette prevalence among college students in these two nations.

**METHODS:**

From May to June 2023, a cross-sectional study was employed to conduct an online questionnaire survey among college students in three universities located in Kunming (China) and Jakarta (Indonesia), respectively. The chi-squared test was utilized to compare the rates/ratios, while binary logistic regression analysis was applied to examine the factors influencing e-cigarette knowledge, attitude, and practice.

**RESULTS:**

A total of 1327 individuals were included in the investigation. The proportion of Indonesian students (75.6%) with a high level of e-cigarette knowledge was lower than that observed among Chinese students (87.4%) (χ^2^=29.7, p<0.001). Additionally, the prevalence of e-cigarette use among Indonesian students (9.4%) was higher compared to their Chinese counterparts (3.0%) (χ^2^=22.32, p<0.001). Binary logistic regression analysis revealed that age, place of residence, studies, gender, and e-cigarette use by friends and family, significantly influenced knowledge levels and attitudes toward e-cigarettes in both countries (p<0.05).

**CONCLUSIONS:**

Despite the positive knowledge, attitudes, and practices towards e-cigarettes among undergraduate students in both countries, a notable knowledge gap exists concerning the harmful effects of e-cigarettes. Chinese students had better e-cigarette knowledge and demonstrated lower usage rates, suggesting that heightened awareness plays a favorable role in preventing e-cigarette use. Furthermore, it becomes imperative for policymakers and health educators to focus on specific factors, such as the influence of close friends and family members, as well as the area of residence.

## INTRODUCTION

Tobacco claims the lives of over 8 million individuals annually, with the majority of these deaths occurring in low- and middle-income nations^1^. Based on data from the World Health Organization (WHO), China and Indonesia are two countries grappling with a high prevalence of smoking worldwide, standing at 26.6% and 34.5% in 2021, respectively^2^. Despite the persistent prevalence of tobacco smokers, electronic cigarettes (e-cigarettes) have gained popularity among young adults globally in recent years. The result of a study by Tehrani et al.^3^ showed the lifetime and current prevalence of e-cigarette vaping among adolescents globally were 23% and 11%, respectively^3^.

E-cigarettes, also known as vapes, are battery-operated devices that deliver nicotine and other substances^4^. Initially designed and marketed as a smoking cessation aid, e-cigarettes have aroused concerns about whether they are able to facilitate smoking cessation or trigger nicotine addiction, as well as the use of flammable products among young people^5-7^. Several studies have confirmed the deleterious effects of e-cigarettes, elucidating the presence of toxic and carcinogenic elements in both the liquid [propylene glycol (PG), diethylene glycol (DG) and nicotine], and the devices (chromium, nickel, and aluminum). These elements have been linked to impaired physiological function in various tissues and organs, resulting in acute and chronic diseases, including chronic obstructive pulmonary disease (COPD), cancers, and asthma^8-10^. Nicotine, in particular, is a highly toxic substance known for its addictive properties and detrimental impact on brain development, affecting the way the synapses are formed, particularly in younger populations^11^.

Although the health hazards of e-cigarettes have been scientifically established, there remains a significant knowledge and attitude gap among young individuals. Studies conducted in China and Indonesia have revealed prevalent misconceptions, with a substantial proportion of young people underestimating the harmful nature of e-cigarettes^12,13^. A study conducted in China found that 62.5% of university students who currently use e-cigarettes believed these products to be less addictive than conventional cigarettes, thereby augmenting their inclination toward using e-cigarettes^14^. Similarly, in Indonesia, 65% of students who currently use e-cigarettes perceived these products as less harmful than conventional cigarettes, which fueled their curiosity to experiment^15^.

E-cigarettes, originally developed by Hon Lik in China, have positioned the country as the global leader in manufacturing, accounting for approximately 80% of the world’s production in 2015^16,17^. Nevertheless, despite its significant role in production, China exhibits a relatively low prevalence of e-cigarette use among adolescents, estimated to range between 0.9% and 1.6%^18^. Recognizing the potential public health implications, the Chinese government has enacted strict national regulations since 2018, as evident in the Healthy China Initiative. This initiative seeks to reduce smoking rates by 20% among individuals aged >15 years through comprehensive measures, including the prohibition of e-cigarette sales to teenagers, removal of e-cigarette advertisements from online platforms, restrictions on flavored e-cigarettes, and the prohibition of manufacturing and dissemination of cigarette-like items such as candies and toys^19,20^.

In contrast, introducing e-cigarettes to the Indonesian market in 2010 marked the commencement of a regulatory landscape characterized by minimal oversight^21^. Consequently, Indonesia exhibits a higher prevalence of e-cigarette use, ranging from 10.7% to 11.8% in Southeast Asian countries^22^. This elevated prevalence can be attributed, in part, to the divergent regulatory approach adopted by Indonesia. Notably, Indonesia has not ratified the Framework Convention on Tobacco Control (FCTC) and maintained unrestricted sales of e-cigarettes. The regulatory landscape is further characterized by the absence of a prohibition on the sale, labeling, flavor, or age restrictions pertaining to e-cigarettes, with the sole anticipated regulatory measure being a projected 15% increase in the excise tax rate on liquid vape products by 2023. The differential regulatory approaches between China and Indonesia underscore the need for comprehensive global strategies to address the burgeoning prevalence of e-cigarette use, considering both production and consumption dynamics^23^.

This is the first comprehensive examination of the knowledge, attitude, and practices associated with electronic cigarette use in adolescents in China and Indonesia. The findings can underscore the need for enhanced regulatory measures and educational programs to mitigate the prevalence of e-cigarette use among undergraduate students in both countries. The disparities identified in both countries will be of considerable importance in providing invaluable, evidence-based insights for researchers, healthcare practitioners, and policymakers, which is paramount to the informed design and implementation of interventions that address the specific needs and challenges associated with e-cigarette use in undergraduates.

## METHODS

### Research design and sampling

Between May and June 2023, we conducted a cross-sectional study utilizing an online self-administered questionnaire among students. The study sites were selected as Kunming, the capital city of Yunnan province in China, and Jakarta in Indonesia due to their comparable prevalence rates of current smoking among adults (33.7% in Yunnan^24^ and 32.2% in Jakarta^25^).

Three universities were randomly selected in each city, comprising one medical university and two non-medical universities from a predetermined list of institutions. Although an initial plan was to employ random sampling, limitations in accessing student enrollment records necessitated adopting a convenience sampling approach, whereby students were conveniently drawn from each university. The selection process ensured a proportional representation of the student population at each chosen institution. The survey targeted undergraduate students aged 18–25 years who were enrolled at their respective universities during data collection.

Previous studies indicated the prevalence of electronic cigarette users at approximately 5.6% in Previous studies indicated the prevalence of electronic cigarette users at approximately 5.6% in Kunming and 5.9% in Jakarta^26,27^. Therefore, the prevalence was set at 0.056 and the allowable error δ takes half of this value, so δ=0.028. Setting efficiency (deff) at 1.5, α at 0.05 for a two-sided test with Z_1-α/2_ equal to 1.96, we calculated the sample size required for this study, which resulted in an estimated minimum sample size of 600 participants per country to be recruited as part of our research efforts.

### Survey measures

To ensure linguistic accessibility, the survey questionnaire was translated into local Chinese and Bahasa Indonesia. It consisted of four sections: basic information, knowledge about e-cigarettes, attitudes towards e-cigarettes, and personal e-cigarette use.

*Basic information*

The initial section focused on gathering demographic data encompassing gender, age, year in school (first to fourth), residence area (urban, rural), monthly living cost (≤300 US$, >300 US$), and whether they had family members or close friends engaged in tobacco or e-cigarette use (yes, no).

*Knowledge*

This section gauged participants’ comprehension of e-cigarettes through nine questions, each utilizing a binary response format (1 for correct and 0 for incorrect responses). A percentage threshold of 75% was established based on previous studies conducted by Aghar et al.^28^ and Bahiru et al.^29^. In our study, participants who correctly answered at least 75% (6/9) of the questions were categorized as having good knowledge, while those scoring below 75% were considered to have poor knowledge^28,29^.

*Attitude*

This section employed a scale incorporating nine items derived from various sources and tailored to our study’s objectives. Questions about attitudes towards e-cigarettes were framed on a binary scale with ‘agree’ assigned a score of ‘0’ and ‘disagree’ assigned a score of ‘1.’ Analogous to the knowledge section, a positive attitude was defined as scoring above 75% on the attitude scale. Participants who answered 75% (6/9) or more questions positively were classified as having a positive attitude, whereas those scoring below 75% were considered negative^28,29^. The positive attitudes encompassed the perception that e-cigarettes do not alleviate stress, lack social acceptability, are not intended for pleasure purposes, and are not considered alternative substitutes for tobacco cigarettes. The total scores were calculated by aggregating all responses indicating a positive attitude, with higher scores reflecting a more favorable standpoint of view.

*Practice*

The final section comprised nine questions assessing the prevalence of e-cigarette use among undergraduate students. Inquiries encompassed current e-cigarette use, smoking duration, frequency of smoking per day, time elapsed before starting to smoke after waking up, expenditure on e-cigarettes, ease of purchase, and preferred purchasing locations. For the item regarding e-cigarette use (yes, no), responses were categorized into two options: ‘currently using’ was scored as ‘1’, while ‘not currently using’ or ‘previously used but not anymore’ was scored as ‘2’.

*Reliability test*

A self-constructed questionnaire was employed as a measuring instrument for data collection. The reliability of the knowledge scales was assessed by conducting a pretest survey in April 2023 involving 20 students per country. The development of the questionnaire followed an ‘adapt-and-adopt’ approach, which was derived from a previous study on e-cigarette knowledge, attitude and practice (KAP)^30^. Content validation procedures were implemented, including a review by experts consisting of public health professionals and epidemiologists. The internal consistency of the questionnaire sections was evaluated using Cronbach’s alpha and Kappa techniques, yielding values of 0.99 and 0.69, respectively.

### Statistical analysis

SPSS (version 27.0) was utilized for analysis. Descriptive statistics were computed, encompassing mean and standard deviation (SD) for quantitative variables, and frequency and percentage (%) for qualitative data. Chi-squared tests were employed to analyze differences in knowledge, attitude, and practice regarding e-cigarettes among undergraduate students from both countries. Bivariate logistic regression analysis was conducted to investigate the influencing factors of e-cigarette knowledge, attitude, and practice with adjusted odds ratios (AORs), 95% confidence intervals (CIs), and a significance level set at p<0.05. The models were adjusted for potential confounding factors, including gender, age, studies, year in school, residence area, living cost, living with smokers, family members using e-cigarettes, and close friends using e-cigarettes.

## RESULTS

### Demographic characteristics

This study enrolled 1327 participants, with 736 (55.5%) females and 591 (44.5%) males. Among the participants, 886 (66.8%) were non-medical students and 441 (33.2%) were medical students. A detailed demographic breakdown is presented in Table 1, revealing no significant differences between medical and non-medical students in both countries.

**Table 1:** Demographic characteristics of university students from May to June 2023 in China and Indonesia (N=1327)

*Characteristics*	*Total (N=1327) n (%)*	*Country*	
		*China (N=625) n (%)*	*Indonesia (N=702) n (%)*
**Sex**			
Male	591 (44.5)	232 (37.1)	359 (51.1)
Female	736 (55.5)	393 (62.9)	343 (48.9)
**Age** (years)			
≤20	1038 (78.2)	435 (69.6)	603 (85.9)
>20	289 (21.8)	190 (30.4)	99 (14.1)
**Studies**			
Medical university	441 (33.2)	208 (33.3)	233 (33.2)
Non-medical university	886 (66.8)	417 (66.7)	469 (66.8)
**Year in School**			
First–Second	1142 (86.1)	565 (90.4)	577 (82.2)
Third–Fourth	185 (13.9)	60 (9.6)	125 (17.8)
**Residence**			
Urban	831 (62.6)	214 (34.2)	617 (87.9)
Rural	496 (37.4)	411 (65.8)	85 (12.1)
**Monthly living cost** (US$)			
≤300	965 (72.7)	538 (86.1)	427 (60.8)
>300	362 (27.3)	87 (13.9)	275 (39.2)
**Smoking tobacco cigarettes**			
Yes	137 (10.3)	52 (8.3)	85 (12.1)
No	1190 (89.7)	573 (91.7)	617 (87.9)
**Currently living with smokers**			
Yes	346 (26.1)	125 (20.0)	221 (31.5)
No	981 (73.9)	500 (80.0)	481 (68.5)
**Family members ever use tobacco cigarettes**			
Yes	298 (22.5)	110 (17.6)	188 (26.8)
No	1029 (77.5)	515 (82.4)	514 (73.2)
**Family members ever use e-cigarettes**			
Yes	79 (6.0)	43 (6.9)	36 (5.1)
No	1248 (94.0)	582 (93.1)	666 (94.9)
**Close friends ever use tobacco cigarettes**			
Yes	752 (56.7)	164 (26.2)	588 (83.8)
No	575 (43.3)	461 (73.8)	114 (16.2)
**Close friends ever use e-cigarettes**			
Yes	387 (29.2)	105 (16.8)	282 (40.2)
No	940 (70.8)	520 (83.2)	420 (59.8)

Among the participants, 8.3% of Chinese students (n=52) and 12.1% of Indonesian students (n=85) reported smoking cigarettes. Furthermore, a substantial majority in both countries indicated that they did not live with smokers: 80.0% for Chinese students and 68.5% for Indonesian students. Regarding close friends who use tobacco cigarettes, the majority of Indonesian students (83.8%) and Chinese students (73%) responded negatively to this question.

### Knowledge about e-cigarettes

A total of 1077 (81.2%) students demonstrated a good level of e-cigarette knowledge. Chinese students exhibited a higher level of knowledge about e-cigarettes (87.4%) compared to Indonesian students (75.6%), with a statistical significance (χ^2^=29.697, p<0.01). Notably, only 68 (5.1%) students were able to correctly answer all questions, with 53 Chinese students (8.5%) and 15 Indonesian students (2.1%), respectively.

The participants’ deficiency in knowledge about e-cigarette smoking became evident through the limited correct responses observed across several questions. Notably, a substantial knowledge gap was observed among the participants, particularly concerning the availability of smoking cessation services. Specifically, more than half of the Chinese students displayed insufficient understanding of the availability of smoking cessation services, with only 240 individuals (32.7%) demonstrating awareness of this crucial topic.

Examining the comprehension of the risk posed by e-cigarettes for cancer, Chinese students exhibited a heightened level of awareness at 50.6%, surpassing their Indonesian counterparts, who demonstrated a recognition rate of 49.4%. Similarly, regarding the perception that e-cigarettes are not less harmful than tobacco cigarettes, Chinese students displayed a higher knowledge level at 52.0%, while Indonesian students presented a corresponding rate of 48.0%. The above findings are summarized in Table 2.

**Table 2:** Knowledge regarding e-cigarettes among undergraduate students from May to June 2023 in China and Indonesia (N=1327)

*Variable*	*Total (N=1327) n (%)*	*Country*		*χ^2^*	*p*
		*China (N=625) n (%)*	*Indonesia (N=702) n (%)*		
**All answered correctly**	68 (5.1)	53 (8.5)	15 (2.1)	27.36	<0.001
**Knowledge level**				29.7	<0.001
Poor	250 (18.8)	79 (12.6)	171 (24.4)		
Good	1077 (81.2)	546 (87.4)	531 (75.6)		
**The questions with the lowest correct response**					
Knowing smoking cessation services	733 (55.2)	240 (32.7)	493 (67.3)	87.33	<0.001
Pose a lower risk for cancer	725 (54.6)	367 (50.6)	358 (49.4)	1.18	0.278
Less harmful than tobacco	766 (57.7)	398 (52.0)	368 (48.0)	0.11	0.738

### Attitude towards e-cigarettes

Our study revealed 87.6% of respondents were favorable toward e-cigarettes, yielding a significant chi-squared value (χ^2^=18.53, p<0.001). Indonesian students demonstrated a higher positive attitude with 641 individuals (91.3%), compared to their Chinese counterparts, where 522 individuals (83.5%) expressed a favorable attitude. A relatively noteworthy percentage of participants displayed a positive attitude towards some aspects of e-cigarettes.

More than half of students in both countries disagreed with the notion that ‘using e-cigarettes can alleviate stress’ (78.6% in China and 88.6% in Indonesia) (χ^2^=24.66, p<0.001).

Exploring the household dynamics, approximately 49.1% of the Chinese respondents reported permitting e-cigarette use within their homes. In contrast, an impressive majority of Indonesian participants (77.4%) expressed a prohibition of such activities within their households. This disparity is underscored by a substantial chi-square value of χ^2^=101.677 and statistical significance at p<0.001.

In terms of governmental regulations on e-cigarette use, both Chinese and Indonesian respondents expressed disagreement and that the government should not regulating the use of e-cigarettes. Specifically, 466 Chinese participants (74.6%) disagreed, while an overwhelming majority (97.6%) of Indonesian students similarly expressed disagreement with this notion (χ^2^=152.28, p<0.001). For further details see Table 3.

**Table 3:** Attitude regarding e-cigarettes among undergraduate students from May to June 2023 in China and Indonesia (N=1327)

*Variable*	*Total (N=1327) n (%)*	*Country*		*χ^2^*	*p*
		*China (N=625) n (%)*	*Indonesia (N=702) n (%)*		
**Gain superiority among my friends**					
Agree	100 (7.5)	59 (9.4)	41 (5.8)	6.15	0.013
Disagree	1227 (92.5)	566 (90.6)	661 (94.2)		
**Give me more pleasure while using it**					
Agree	145 (10.9)	66 (10.6)	79 (11.3)	0.16	0.686
Disagree	1182 (89.1)	559 (89.4)	623 (88.7)		
**Relieve stress after using e-cigarettes**					
Agree	214 (16.1)	134 (21.4)	80 (11.4)	24.66	<0.001
Disagree	1113 (83.9)	491 (78.6)	622 (88.6)		
**E-cigarettes make me feel fit and cool**					
Agree	163 (12.3)	106 (17.0)	57 (8.1)	23.98	<0.001
Disagree	1164 (87.7)	519 (83.0)	645 (91.9)		
**Socially acceptable to smoke e-cigarettes**					
Agree	276 (20.8)	196 (31.4)	80 (11.4)	80	<0.001
Disagree	1051 (79.2)	429 (68.6)	622 (88.6)		
**Allow people to smoke e-cigarettes in your home**					
Agree	466 (35.1)	307 (49.1)	159 (22.6)	101.68	<0.001
Disagree	861 (64.9)	318 (50.9)	543 (77.4)		
**Someone who uses e-cigarettes is not a smoker**					
Agree	203 (15.3)	183 (29.3)	20 (2.8)	178.26	<0.001
Disagree	1124 (84.7)	442 (70.7)	682 (97.2)		
**Government should not regulate the use of e-cigarettes**					
Agree	176 (13.3)	159 (25.4)	17 (2.4)	152.28	<0.001
Disagree	1151 (86.7)	466 (74.6)	685 (97.6)		
**E-cigarettes for pleasure**					
Agree	145 (10.9)	118 (18.9)	27 (3.8)	76.78	<0.001
Disagree	1182 (89.1)	507 (81.1)	675 (96.2)		
**Attitude level**					
Negative	164 (12.4)	103 (16.5)	61 (8.7)	18.53	<0.001
Positive	1163 (87.6)	522 (83.5)	641 (91.3)		

**Figure 1:**
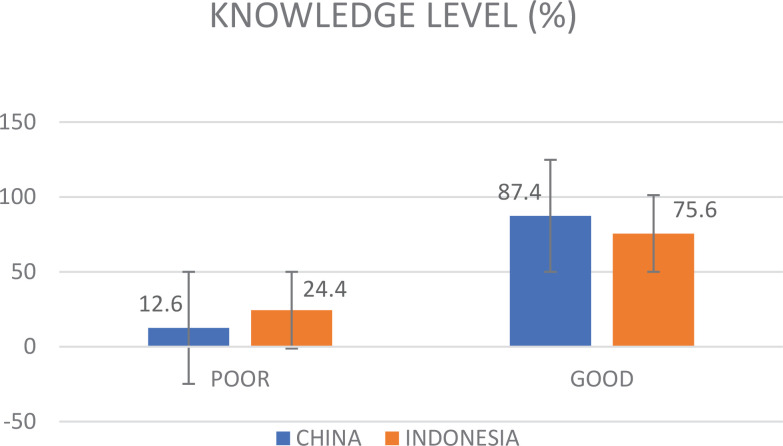
Knowledge level among undergraduate students from May to June 2023 in China and Indonesia (N=1327)

### E-cigarette practice

The prevalence of e-cigarette use in Indonesia (9.4%) was found to be higher than that in China (3%). Examining the duration of use within the last seven days, a notable proportion of Indonesian students (56.1%) reported using e-cigarettes for more than five days, while the majority of Chinese students (63.2%) reported only 1–2 days. Both countries demonstrated high frequencies of e-cigarette usage per day, with 47.4% of Chinese students and 68.2% of Indonesian students reporting a use of <20 times a day. Furthermore, over half of the participants in both countries purchased their e-cigarettes from authentic stores (73.7% in China and 81.8% in Indonesia). Notably, respondents in Kunming perceived the purchase of e-cigarettes as challenging (63.2%), whereas the majority in Indonesia found it easy (86.4%).

Concerning expenditure, approximately half of the Chinese students spent between 14–28 US$ on each purchase, while almost half of the Indonesian students spent <14 US$ per purchase. Regarding age at first try, individuals who first experimented with e-cigarettes predominantly occurred before the age of 18 years. Additionally, a significant proportion expressed no desire to quit using e-cigarettes, with 52.6% of students from China and 85.7% from Indonesia falling into this category. Detailed information regarding these findings is presented in Table 4.

**Table 4:** Practice regarding e-cigarettes among undergraduate students from May to June 2023 in China and Indonesia (N=1327)

*Variable*	*Total (N=1327) n (%)*	*Country*		*χ^2^*	*p*
		*China (N=625) n (%)*	*Indonesia (N=702) n (%)*		
**Currently using e-cigarettes**					
Yes	85 (6.4)	19 (3)	66 (9.4)	22.32	<0.001
No	1242 (93.6)	606 (97)	636 (90.6)		
**Number of days used within the last 7 days**					
1–2	20 (23.5)	12 (63.2)	8 (12.1)	21.42	<0.001
3–4	24 (28.3)	3 (15.8)	21 (31.8)		
≥5	41 (48.2)	4 (21)	37 (56.1)		
**Frequency of e-cigarette use per day**					
Not daily	9 (10.6)	5 (26.3)	4 (6.1)	6.72	0.035
<20	54 (63.5)	9 (47.4)	45 (68.2)		
≥20	22 (25.9)	5 (26.3)	17 (25.7)		
**Time of initiating use after waking-up**					
Immediately after waking-up	19 (22.4)	8 (42.1)	11 (16.7)	5.88	0.053
After 1–2 hours	42 (49.4)	8 (42.1)	24 (51.5)		
It varies	24 (28.2)	3 (15.8)	21 (31.8)		
**Place of purchase of e-cigarettes**					
Online shop	17 (20)	5 (26.3)	12 (18.2)	0.61	0.435
Authentic store	68 (77.6)	14 (73.7)	54 (81.8)		
**Ease of purchase**					
Yes	64 (75.3)	7 (36.8)	57 (86.4)	19.45	<0.001
No	21 (24.7)	12 (63.2)	9 (13.6)		
**Price per purchase** (US$)					
<14	38 (44.7)	6 (31.6)	32 (48.5)	2.51	0.285
14–28	28 (32.9)	9 (47.4)	19 (28.8)		
>28	19 (22.4)	4 (21)	15 (22.7)		
**Price of e-cigarette liquid** (US$)					
<4	50 (58.8)	9 (47.4)	41 (62.1)	1.33	0.515
4–5	21 (24.7)	6 (31.6)	15 (22.7)		
>5	14 (16.5)	4 (21.1)	10 (15.2)		
**Age of first try** (years)					
<18	58 (68.2)	9 (47.4)	49 (74.2)	4.92	0.027
≥18	27 (31.8)	10 (52.6)	17 (25.8)		
**Intention to quit**					
Yes	12 (14.1)	9 (47.4)	3 (4.5)	22.31	<0.001
No	73 (85.9)	10 (52.6)	63 (95.5)		

### Factors associated with knowledge, attitude, and practice towards e-cigarettes

Binary logistic regression analysis was conducted to identify significant factors, incorporating three independent variables (knowledge, attitude, and practice) into the logistic regression models. The models were adjusted for potential confounding factors, including gender, age, studies, year in school, residence area, living cost, living with smokers, family members using e-cigarettes, and close friends using e-cigarettes. The same regression model was applied in both countries, and only the model variables that achieved significance after controlling for all covariates are presented in Table 5.

**Table 5:** Logistic regression analyses association of sociodemographic factors with knowledge, attitude and practice of e-cigarettes among university students from May to June 2023 in China and Indonesia (N=1327)

*Variable*	*Influencing factors*	*Total AOR (95% CI)*	*Country*	
			*China AOR (95% CI)*	*Indonesia AOR (95% CI)*
**Knowledge** (Poor vs Good)	Medical (Ref: Non-medical)	1.72 (1.277–2.321)**		3.57 (2.36–5.41)**
	Living with smokers (Ref: No)	2.20 (1.536–3.165)**		3.25 (2.09–5.07)**
	Third–fourth years (Ref: First–Second years)			0.45 (0.22–0.93)*
	Close friends use e-cigarettes (Ref: No)	1.39 (1.030–1.894)*		
**Attitude** (Positive vs negative)	>20 years old (Ref: ≤20 years old)	0.61 (0.385–0.968)*		
	Rural (Ref: Urban)	0.68 (0.473–0.993)*		
	Medical (Ref: Non-medical)		0.59 (0.36–0.99)*	
	Living with smokers (Ref: No)	2.43 (1.583–3.736)**		5.29 (2.71–10.32)**
**Practice** (Use vs Not use)	Male (Ref: Female)	3.07 (1.926–4.914)**	3.82 (1.95–7.5)**	2.96 (1.49–5.91)*
	Medical (Ref: Non-medical)		0.31 (0.19–0.73)*	
	Living with smokers (Ref: No)	3.49 (2.24–5.43)**	2.34 (1.15–4.8)*	5.40 (2.92–10.02)**
	Family members use e-cigarettes (Ref: No)	2.98 (1.71–5.2)**	4.05 (1.67–9.86)*	2.60 (1.17–5.79)*

AOR: adjusted odds ratio.

* p<0.05,

** p<0.01.

Students who did live with smokers displayed better knowledge about e-cigarettes compared to their counterparts not living with smokers (AOR=2.20; 95% CI: 1.536–3.165). However, students with more favorable attitudes towards e-cigarettes (AOR=2.43; 95% CI: 1.58–3.74) were likely to use e-cigarettes (AOR=3.49; 95% CI: 2.24–5.43). Moreover, medical students exhibited a better knowledge of e-cigarettes when compared to non-medical students (AOR=1.72; 95% CI: 1.28–2.32).

Distinguishing factors influencing e-cigarette knowledge and attitudes exhibited notable variations between the two countries. Chinese medical students exhibited a positive attitude towards the harm caused by e-cigarettes compared with non-medical students (AOR=0.596; 95% CI: 0.358–0.992). A distinctive pattern was observed among medical students in China, revealing a lower likelihood of engaging in e-cigarette use (AOR=0.31; 95% CI: 0.189–0.730), while those living with people who used e-cigarettes were more likely to adopt such behavior themselves (AOR=2.34; 95% CI : 1.15–4.8).

In Indonesia, medical students demonstrated substantial knowledge regarding e-cigarettes (AOR=3.57; 95% CI: 2.36–5.41). Students who lived with smokers displayed better knowledge about e-cigarettes compared to their counterparts not living with smokers (AOR=3.25; 95% CI: 2.09–5.07) and displayed a more positive attitude towards the harm caused by e-cigarettes (AOR=5.29; 95% CI: 2.71–10.32). However, they had an increased likelihood of using e-cigarettes themselves (AOR=5.40; 95% CI: 2.92–10.02). Lastly, Indonesian students in their third–fourth years possessed considerable knowledge about e-cigarettes (AOR=0.45; 95% CI: 0.22–0.93). All statistically significant results are presented in Table 5.

## DISCUSSION

E-cigarettes, marketed as a potential harm reduction strategy for tobacco cigarette smoking, raise concerns regarding safety and efficacy, with limited knowledge of their health effects^31^. Given the escalating prevalence of e-cigarette use among young undergraduate students, this study aimed to investigate the knowledge, attitudes and practices pertaining to e-cigarettes in Kunming (China) and Jakarta (Indonesia).

Our findings highlight the need for students in both countries to receive evidence-based education on e-cigarettes. The study identified variations in e-cigarette knowledge between Indonesia (75.6%) and China (87.4%), surpassing rates reported in previous studies in Hangzhou, China (42.6%)^32^, and in Indonesia (37.9%)^33^. This discrepancy may stem from potential differences in health literacy, particularly among the surveyed medical students. Despite generally good knowledge, a substantial portion of students lacked awareness of smoking cessation services (55.2%) and believed e-cigarettes posed a lower cancer risk (54.6%). Additionally, a significant proportion perceived e-cigarettes as safer than traditional cigarettes (57.7%), emphasizing insufficient comprehension of associated health risks. This is consistent with previous research conducted by Canzan et al.^24^, which revealed that students who were not exposed to information about e-cigarettes were more likely to consume these products due to a lack of knowledge of their chemical makeup^34^.

The prevalence of e-cigarette use varied between countries, with Chinese students exhibiting lower rates compared to Indonesian students, possibly influenced by nationwide regulations. China’s stringent regulations, including flavor bans and high taxes, contrast with Indonesia’s limited regulations, contributing to the observed differences. Specifically, the Chinese government has implemented regulations on e-cigarettes, such as banning flavored varieties other than tobacco flavor domestically and selling through vending machines and online platforms. Additionally, the Chinese government imposes high taxes on e-cigarettes^19,20^. On the contrary, in Indonesia, there are no specific regulations regarding e-cigarettes except for an anticipated 15% increase in the excise tax rate on liquid vape by 2023^23^.

Positive attitudes toward e-cigarette use were more prevalent among Indonesian students (91.3%) compared to Chinese students (83.5%), indicating a shift from prior studies. Consistent with existing literature, widespread support for government regulation emphasizes the need for clear policies on e-cigarette use. For instance, Aghar et al.^28^ reported that 70% of respondents believed that governmental regulations should be imposed on e-cigarettes. Our findings have revealed a significant association between e-cigarette use and factors such as gender, smoking status of friends, cohabitation with smokers, and urban residence. The influence of social factors is crucial in shaping adolescent e-cigarette use, particularly due to their susceptibility to peer and familial influences^35^. Moreover, urban students who are exposed to social media and have increased accessibility exhibit a higher propensity for experimenting with e-cigarettes. These findings emphasize the need to consider these factors in designing effective intervention strategies^36^.

The study emphasizes the urgent need for policymakers, particularly in Indonesia, to establish clear regulations and educational programs targeting undergraduate students to curb the rising prevalence of e-cigarette use. Despite participants’ good knowledge and positive attitudes, the alarming increase in usage in Indonesia calls for immediate action. Disparities with prior studies may stem from variations in study populations, as our focus on medical and non-medical students differs from previous research on the general population. Recommendations include tailored educational interventions and regulatory measures to address the evolving landscape of e-cigarette use among young individuals.

### Limitations

There are four primary limitations in this study. To enhance the comprehensiveness of the current understanding of e-cigarette use, future surveys should encompass a broader range of universities in both countries to capture a more representative sample of the target population. Secondly, longitudinal data should be considered to establish causal relationships between variables and explore potential confounding factors further. Thirdly, it is essential to conduct extensive research on gender-specific factors and attitudes towards e-cigarette use as they can significantly influence behaviors. Finally, despite employing representative sampling techniques and rigorous quality control throughout the project design and implementation process, incorporating stratified random sampling with precise calculations for sample weights would improve accuracy.

## CONCLUSIONS

Despite the positive knowledge, attitudes, and practices towards e-cigarettes among undergraduate students in both countries, a notable knowledge gap exists concerning the harmful effects of e-cigarettes. This gap is consistent with findings from analogous studies, indicating a widespread lack of understanding among participants about the potential risks associated with e-cigarette use. While the current research reveals positive attitudes toward e-cigarettes, a significant proportion of participants still perceive these products as posing a lower risk for cancer and consider them to be safer when compared to traditional cigarettes. Furthermore, disparities in e-cigarette practices are evident, with Indonesia exhibiting a higher prevalence compared to China. In light of these findings, it becomes imperative for policymakers and health educators to focus on specific factors, such as the influence of close friends and family members, as well as the area of residence. Targeting these factors in preventive measures is crucial for controlling the consequences associated with e-cigarette use. This study underscores the necessity for evidence-based education regarding e-cigarettes among students in both countries. Initiating awareness campaigns among students is a crucial first step toward reducing the incidence rate within the healthcare system. Additionally, there is a pressing need for governmental facilitation and promotion of regulations pertaining to e-cigarettes. This is especially pertinent for the Indonesian government, emphasizing the importance of establishing clear regulations on e-cigarettes to safeguard public health.

## Data Availability

Data sharing is not applicable to this article as no new data were created.
